# Setting occupational exposure limits for antimicrobial agents: A case study based on a quaternary ammonium compound-based disinfectant

**DOI:** 10.1177/0748233720970438

**Published:** 2020-11-26

**Authors:** G Scott Dotson, Jason T Lotter, Rachel E Zisook, Shannon H Gaffney, Andrew Maier, Jonathan Colvin

**Affiliations:** 1 205740 Cardno ChemRisk, Cincinnati, OH, USA; 2 205740 Cardno ChemRisk, Chicago, IL, USA; 3 205740 Cardno ChemRisk, San Francisco, CA, USA; 4 Drug and Poison Information Center, 2518 Cincinnati Children’s Hospital Medical Center, Cincinnati, OH, USA

**Keywords:** Antimicrobial agents, occupational exposure limits, disinfectants, workplace, risk assessment

## Abstract

Antimicrobial agents have become an essential tool in controlling the transmission of severe acute respiratory syndrome coronavirus 2 (SARS-CoV-2), and guidelines on their use have been issued by various public health agencies. Through its *Emerging Viral Pathogen Guidance for Antimicrobial Pesticides*, the US Environmental Protection Agency has approved numerous surface disinfectant products for use against SARS-CoV-2. Despite their widespread use and range of associated health hazards, the majority of active ingredients in antimicrobial products, such as surface disinfectants, lack established occupational exposure limits (OELs) to assist occupational health professionals in characterizing risks from exposures to these chemicals. Based on established approaches from various organizations, a framework for deriving OELs specific to antimicrobial agents was developed that relies on a weight-of-evidence evaluation of the available data. This framework involves (1) a screening-level toxicological assessment based on a review of the existing literature and recommendations, (2) identification of the critical adverse effect(s) and dose–response relationship(s), (3) identification of alternative health-based exposure limits (HBELs), (4) derivation of potential OELs based on identified points of departure and uncertainty factors and/or modification of existing alternative HBELs, and (5) selection of an appropriate OEL. To demonstrate the use of this framework, a case study is described for selection of an OEL for a disinfectant product containing quaternary ammonium compounds (quats). Three potential OELs were derived for this product based on irritation toxicity data, developmental and reproductive toxicity (DART) data, and modification of an existing HBEL. The final selected OEL for the quats-containing product was 0.1 mg/m^3^, derived from modification of an existing HBEL. This value represented the lowest resulting value of the three approaches, and thus, was considered protective of irritation and potential DART.

## Introduction

In response to the emergence of severe acute respiratory syndrome coronavirus 2 (SARS-CoV-2), the virus responsible for COVID-19, products containing antimicrobial agents have received increased attention for their role in reducing the transmission of the virus. In March 2020, following the identification of COVID-19 as an emerging pathogen outbreak by the Centers for Disease Control and Prevention, the US Environmental Protection Agency (EPA) issued *List N: Disinfectants for Use Against SARS-CoV-2* (EPA, 2020a). This list initially identified approximately 250 surface disinfectants that met the EPA’s criteria for efficacy under the *Emerging Viral Pathogen Guidance for Antimicrobial Pesticides* (EPA, 2016, 2020a). As of August 20, 2020, *List N* included 482 surface disinfectants meeting the EPA’s criteria for efficacy against SARS-CoV-2 (EPA, 2020a). Approximately 81% (*n* = 389) of the disinfectants contained a single active ingredient, with the remaining disinfectants being composed of mixtures of two or more active ingredients. Of the single active ingredient disinfectants, approximately 48% contained quaternary ammonium compounds (quats), while sodium hypochlorite (bleach) and hydrogen peroxide represented approximately 19% and 11%, respectively. The remaining single active ingredient disinfectants (approximately 22%) contained organic and inorganic acids, chlorinated compounds, aldehydes, alcohols, phenolic, or silver ions.

The properties of antimicrobial agents, such as those included in *List N*, may cause a wide range of adverse health effects in exposed individuals under certain conditions. For example, the majority of the active ingredients are point of contact irritants (i.e. eyes, skin, and respiratory tract) and some may be corrosive in concentrated form. Additionally, these active ingredients may contribute to the onset of acute systemic effects (e.g. central nervous system depression) or repeated-dose effects, such as developmental and reproductive toxicity (DART). Due to concerns regarding occupational exposures to disinfectants, the EPA has requested that the National Toxicology Program (NTP) investigates the link between respiratory illnesses and worker exposures for 10 selected antimicrobial active ingredients (NTP, 2019a). Further, the majority of the active ingredients under investigation by NTP are components of the disinfectants included in *List N.*

Despite being widely recognized as potential occupational hazards, the overwhelming majority of antimicrobial agents, including those found on *List N*, do not have occupational exposure limits (OELs). As illustrated by Table 1, only three of the antimicrobial agents under investigation by NTP have OELs issued by the Occupational Safety and Health Administration (OSHA), National Institute for Occupational Safety and Health (NIOSH), the American Conference of Governmental Industrial Hygienists (ACGIH), or Occupational Alliance for Risk Science-Workplace Environment Exposure Levels (OARS-WEEL) Committee. Further, no OELs were identified for quats, which represent nearly half of the disinfectants included in *List N*. With increased use of antimicrobial agents in response to SARS-CoV-2, there is a need for health-based OELs to aid in protecting the safety and health of workers who may routinely encounter these chemicals in occupational settings.

**Table 1.** OELs for active ingredients of antimicrobial products under investigation by the National Toxicology Program (NTP 2019a).

Common name (chemical name)	CAS #	ACGIH (2019)	NIOSH (2020)	OSHA (2020)	OARS-WEEL (2020)
Alkyl dimethyl benzyl ammonium chloride (ADBAC QUAT)	68424-85-1	None	None	None	None
Bleach (sodium hypochlorite)	7681-52-9	None	None	None	None
Chlorinated isocyanurate (trichloro-*s*-triazinetrione)	87-90-1	None	None	None	None
Chlorine dioxide in solution (chlorine dioxide)	10049-04-4	TLV TWA (0.1 ppm) TLV STEL (0.3 ppm)	REL TWA (0.1 ppm)	PEL TWA (0.1 ppm) PEL STEL (0.3 ppm)	None
1-Decanaminium, *N*-decyl-*N*, *N*-dimethyl chloride (DDAC QUAT)	7173-51-5	None	None	None	None
Hydrogen peroxide (hydrogen peroxide)	7722-84-1	TLV TWA (1 ppm)	REL TWA (1 ppm)	PEL TWA (1 ppm)	None
OPP	90-43-7	None	None	None	None
PAA	79-21-0	TLV STEL (0.4 ppm)	None	None	None
PCMC	59-50-7	None	None	None	None
PHMB	32289-58-0	None	None	None	None

PHMB: poly(hexamethylene biguanide) hypochloride; PCMC: *p*-chloro-*m*-cresol; PAA: peracetic acid; OPP: ortho phenyl phenol; OEL: occupational exposure limit; ACGIH: American Conference of Governmental Industrial Hygienist; CAS #: chemical abstract number; NIOSH: National Institute for Occupational Safety and Health; OSHA: Occupational Safety and Health Administration; PEL: permissible exposure limit; REL: recommended exposure limit; STEL: short-term exposure limit (15 min); TLV: threshold limit value; TWA: time weighted average; OARS-WEEL: Occupational Alliance for Risk Science-Workplace Environmental Exposure Level.

The objective of this manuscript is to present a framework for the derivation of OELs for antimicrobial agents. This framework is based on the current state-of-the-science for setting OELs and relies on a weight-of-evidence approach to assist in the scientific interpretation of the available data. A case study highlights the application of the framework and the development of an OEL for a quat-based disinfectant selected from *List N*.

## Methods

OELs are health-based benchmarks and one of the primary tools relied upon by occupational health professionals to characterize risks of chemical exposures potentially encountered by workers (Deveau et al., 2015; Maier et al., 2015; Nielsen and Ovrebo, 2008; NIOSH, 2019a; Waters et al., 2015). These benchmarks are developed by numerous governmental agencies and professional associations, such as ACGIH, California OSHA, German Research Foundation (DFG), NIOSH, OARS-WEEL Committee, and OSHA. In addition, multiple approaches for setting OELs have been published for specific chemical classes and health endpoints (Deveau et al., 2015, 2017; Dotson et al., 2015; ECHA, 2008; ECETOC, 2014; Frank et al., 2019; Nielsen and Ovrebo, 2008; Schenk and Johanson, 2010). Regardless of the organization or approach, the OEL development process typically incorporates the following components:

1. Critical review of available scientific data to identify potentially relevant studies;
2. Characterization of the hazard or the potential adverse effects associated with a specific chemical;
3. Identification of the critical adverse effect(s);
4. Dose–response assessment for critical adverse effect(s) and identification of the point(s) of departure (PoD);
5. Selection and application of uncertainty factors (UF) based on the quality of the study, severity of observed effects, data insufficiencies, and nature of the PoD;
6. Calculation of potential OEL(s) by dividing the PoD by UF (PoD/UF); and
7. Selection of final OEL based on total weight-of-evidence.

**Figure 1.**
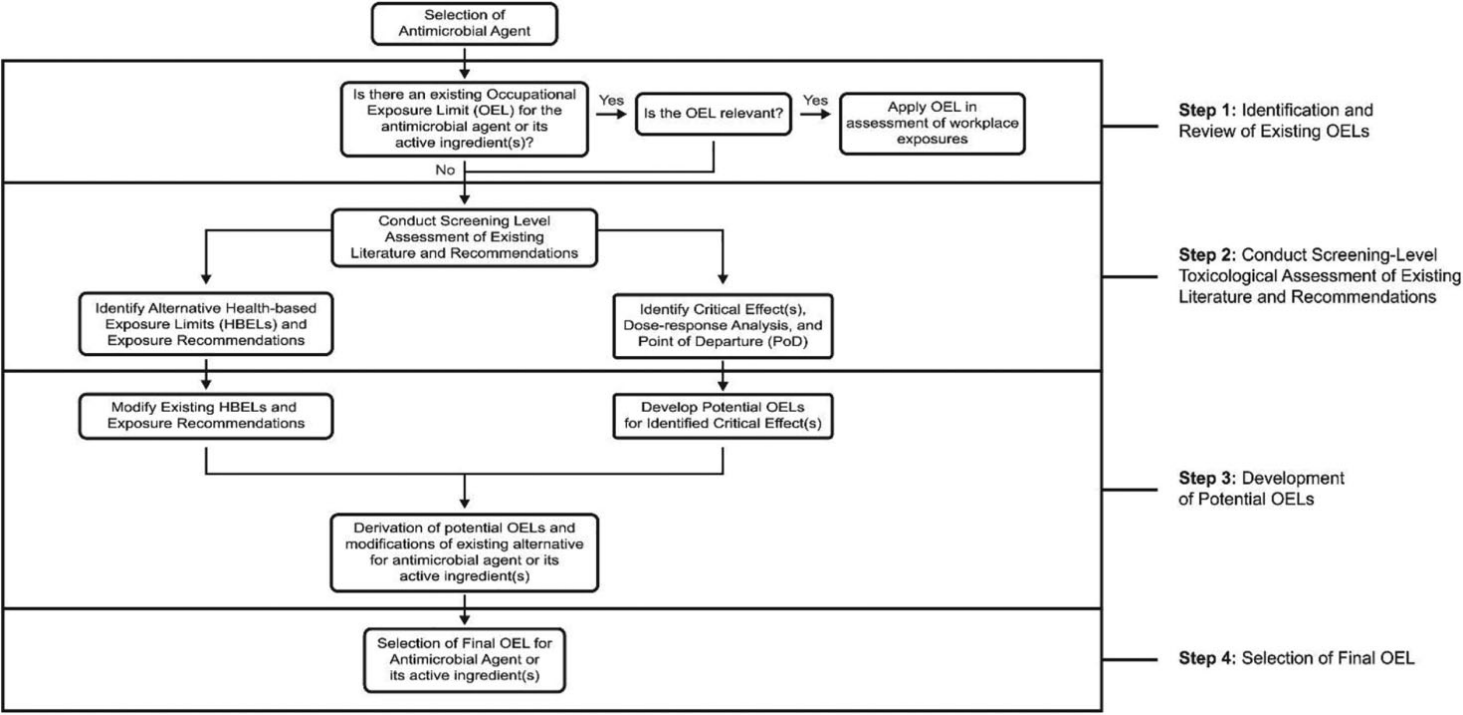
Framework for the derivation of OELs for antimicrobial agents. OEL: occupational exposure limit.

Based on the previously outlined process and the current state-of-the-science for OEL setting, a framework for deriving OELs for antimicrobial agents was developed and is outlined as a flowchart in Figure 1. The application of this framework in a case study based on an undisclosed quat-based disinfectant product selected from *List N* is described below. This case study describes critical considerations made during each of the primary steps of the derivation process and demonstrates the calculation of potential OELs based on different identified potential outcomes of interest or lines of evidence.

## Case study

A review of the quat-based disinfectant product information and safety data sheet (SDS) revealed that it had not been specifically tested against SARS-CoV-2 but was approved for inclusion on *List N* based on the criteria outlined in the *Emerging Viral Pathogen Guidance for Antimicrobial Pesticides* (EPA, 2016). The SDS identified four quats and ethanol as active ingredients that, in total, represented over 6% of the concentrated solution; the remainder of the product (<94%) was described as containing proprietary inert ingredients. Table 2 provides supplemental information on the active ingredients and their relative concentrations within the concentrated solution of the product.

**Table 2.** Active ingredients of antimicrobial product selected from the EPA *List N*.

Active ingredient	CAS #	Relative concentration in concentrated solution
ADBAC	68424-85-1	>2.0%
ODDAC	32426-11-2	∼2.0%
Dio-DAC	5538-94-3	∼1.0%
DDAC	7173-51-5	∼1.0%
Ethanol	64-17-5	<1.0%

ADBAC = alkyl (C_12_-C_16_) dimethyl benzyl ammonium chloride; ODDAC: octyl decyl dimethyl ammonium chloride; Dio-DAC: dioctyl dimethyl ammonium chloride; DDAC: didecyl dimethyl ammonium chloride; CAS #: chemical abstract service number.

### Step 1: Identification and review of existing OELs

The first step following the selection of the disinfectant and review of its product information (i.e. SDS) is to determine whether OELs exist for the active ingredients included in Table 2. This review yielded no OELs for the overall disinfectant or any of the quats. Several OELs were identified for ethanol and are summarized in Table 3. Among these OELs, the documentation for the ACGIH threshold limit value (TLV) short-term exposure limit (STEL) was the most in-depth and provided information on the rationale associated with the recommendation. ACGIH (2009) reported that short-term exposures to ethanol vapor resulted in upper respiratory tract and eye irritation at >1000 ppm in animals, while long-term exposures to concentrations exceeding 10,000 ppm may result in liver cirrhosis, developmental changes in offspring, and potential fertility issues. Based on this information, ACGIH (2009) established a TLV-STEL of 1000 ppm with upper respiratory tract irritation being identified as the critical, acute effect of exposure to ethanol vapor. Further, it was stated that the TLV-STEL is anticipated to provide protection from not only upper respiratory tract irritation but also other effects including the previously described long-term effects because they occur at concentrations well above 1000 ppm.

**Table 3.** Occupational exposure limits for ethanol.

Chemical	CAS #	ACGIH (2009)	OSHA (2020)	NIOSH (2020)	DRG (2019)	Cal/OSHA (2020)	OARS-WEEL Committee (2020)
Ethanol	64-17-5	1000 ppm (TLV-STEL)	1000 (PEL TWA)	1000 (REL TWA)	200 ppm (MAK-TWA) 800 ppm (15-min)	1000 (PEL TWA)	None

ACGIH: American Conference of Governmental Industrial Hygienists; Cal/OSHA: California Occupational Safety and Health Administration; CAS #: chemical abstract number; DRG: German Research Foundation; MAK: maximum airborne concentration; NIOSH: National Institute for Occupational Safety and Health; OSHA: Occupational Safety and Health Administration; PEL: permissible exposure limit; REL: recommended exposure limit; STEL: short-term exposure limit; TWA: 8-h time-weighted average; OARS-WEEL: Occupational Alliance for Risk Science-Workplace Environmental Exposure Level.

In addition, the SDS for the selected disinfectant identified skin corrosion/irritation and serious eye damage/eye irritation as health effects of concern, which was supported by the following Globally Harmonized System of Classification and Labeling of Chemicals (GHS) hazard statements: *H314—Causes severe skin burns and eye damage* and *H318—Causes serious eye damage*. No additional information was identified in the SDS or additional product information regarding systemic toxic effects, such as neurotoxicity, cancer, or DART. As noted above, the documentation for the TLV-STEL for ethanol reports that the primary health concern is upper respiratory irritation; there was no indication of skin burns, corrosion, or serious eye damage (ACGIH, 2009). Therefore, it is unlikely that the hazards of ethanol are the basis for the product warnings or GHS hazard statements. In addition, the quats represent a larger portion of the concentrated solution than ethanol and are generally recognized for their ability to cause skin corrosion and eye damage under certain conditions (Luz et al., 2020). Based on the available evidence, the quats were identified as being the basis for the recognized health effects listed on the SDS (i.e. skin corrosion and serious eye damage).

### Step 2: Conduct screening-level toxicological assessment of existing literature and recommendations

The next step in the framework focuses on conducting a screening-level assessment of the available toxicological data for the quats. There are various levels of depth for conducting a systematic review that can be applied in a fit-for-purpose approach (EPA, 2018; NTP, 2019b). To facilitate the assessment, a literature search of numerous databases should be implemented to identify relevant toxicological data on the antimicrobial agent and its active ingredients. Databases reviewed in the literature search may include but are not limited to PubChem (NLM, 2020a), PubMed (NLM, 2020b), EPA ChemView database (EPA, 2020b), and European Chemical Agency (ECHA’s) Registration, Evaluation, Authorization and Restriction of Chemicals database (ECHA, 2020a).

The principles of literature identification included in these approaches were applied to conduct the screening level assessment of available data for this case study (i.e. quats listed as active ingredients for the selected disinfectant). The initial literature search via PubMed was conducted using multiple combinations of search terms including, but limited to, “quaternary ammonium compounds,” “quats,” “disinfectants,” “antimicrobial,” “toxicity,” and “health effects,” in addition to the names of the individual quats and CAS numbers. The results of these searches yielded over 1000 potential studies. As such, subsequent reviews of EPA (2020b), ECHA (2020a), and PubChem (NLM, 2020a) databases were conducted for the individual quats to aid in refining the literature search strategy. Overall, the assembled database was limited and all studies were evaluated using a weight-of-evidence approach considering human and scenario relevance. It should be noted that if the database was robust, a formal assessment of data quality would have been conducted in accordance with systemic review principles.

PubChem (NLM, 2020a) provided limited useful information, but EPA (2020b) and ECHA (2020a) provided toxicological data for all of the quats other than octyl decyl dimethyl ammonium chloride (ODDAC). Limited, or no data, were available in the ECHA database for repeated exposures, carcinogenicity, or sensitization. Several DART studies based on standard test guidelines (or equivalent) using rat and rabbit models were identified within ECHA (2020a). Based on the information obtained from ECHA (2020a), additional searches of PubMed (NLM, 2020b) were conducted using the previously identified terms in addition to “reproductive,” “developmental,” “carcinogenicity,” and “sensitization.” The refined searches yielded a peer-reviewed hazard review of alkyl (C12-C16) dimethyl benzyl ammonium chloride (ADBAC) and didecyl dimethyl ammonium chloride (DDAC) (Luz et al., 2020), in addition to studies investigating DART in rodents exposed to quat-based disinfectants composed of a mixture of ADBAC and DDAC (Melin et al., 2014, 2016). The searches also resulted in identification of EPA technical reports for ADBAC and DDAC (EPA, 2006a, b). Information on the other quats was not located.

### Step 2a: Identification of critical effect and dose–response analysis

ECHA (2020a), EPA (2006a, b), and Luz et al. (2020) provided the most comprehensive critiques of the toxicological data. The following summarizes the key findings on the toxicity of quats:

- The quats exhibit a low potential for dermal absorption (ECHA, 2020b, c, d; EPA, 2006a, b; Luz et al., 2020).
- The primary health hazards of concern were severe irritation and corrosion of the skin and eyes.

  ∘  ECHA (2020b, c, d) provided evidence of skin corrosion, necrosis, and severe irritation in a dose–response manner following a single application of various solutions. These solutions ranged from undiluted to 50% quat solutions.
  ∘  Ocular studies reported that eye irritation occurred in a dose–response manner following the application of a single dose of quats (ECHA, 2020b, c, d).

- Several repeated exposure (i.e. subchronic and chronic) studies via multiple exposure routes were identified. Overall, these studies did not support a link between exposures to quats and systemic toxicity, such as neurotoxicity, or hematological effects (ECHA, 2020b, c, d; Luz et al., 2020).
- Chronic oral studies and in vitro studies investigating cancer or genotoxicity were identified. The results of these studies were negative and did not provide evidence of carcinogenicity or genotoxicity associated with the quats (ECHA, 2020b, c, d; Luz et al., 2020).

The data regarding an association between quats and DART are not as clear as the data regarding other toxicological endpoints, such as point of contact effects (i.e. irritation and corrosion), systemic effects, or carcinogenicity. DART studies of various designs are available in the ECHA dossiers for ADBAC, Dio-DAC, and DDAC (ECHA, 2020b, c, d). Data from studies using standard test guidelines (or equivalent) for DART in rats and rabbits have shown either no evidence of DART or DART effects that occur at the same or higher doses as paternal effects. Additionally, quats have not been classified by the EPA, ECHA, NTP, California Proposition 65, or other organizations as DART based on the overall body of evidence (CA OEHHA, 2020a; ECHA, 2020b, c; EPA, 2020c; NTP, 2020). In contrast, Melin et al. (2014, 2016) reported a decrease in fertility in both male and female mice exposed to a disinfectant mixture containing approximately 6.8% ADBAC and 10.1% DDAC. In these studies, female and male mice were exposed via their food or water to a mixture of ADBAC+DDAC at 0, 60, or 120 mg/kg/day for varying durations ranging from 2 weeks to 180 days. Melin et al. (2014) reported clinical signs of toxicity, in addition to other effects, such as inappetence, lethargy, and rough hair coat, at 60 mg/kg/day. Further, the authors reported a reduction of the average number of pregnancies and decrease in the cumulative number of live pups born in the 120-mg/kg/day dose group. No effects on fertility or development were reported in the mice treated at 60 mg/kg/day. Luz et al. (2020) stated that the results of Melin et al. (2014) support a no-observed-adverse-effect level (NOAEL) of 60 mg/kg/day for effects on fertility and a lowest-observed-adverse-effect level (LOAEL) of 60 mg/kg/day for maternal toxicity. Melin et al. (2016) reported that the quats caused decreased reproductive capacity in the female mice, including reduced ovulation and fewer estrus cycles. Further, the study reported that male mice exhibited significantly decreased sperm concentration and motility. Luz et al. (2020) reported that the LOAEL for this study would be 120 mg/kg/day for fertility effects. Overall, Melin et al. (2014, 2016) raised a concern regarding an association between exposure to quats and DART but did not provide conclusive evidence as to whether the quats can induce such effects or the potential mechanisms involved. The lack of mechanistic indicators of DART toxicity and the well-described effects of paternal health on developmental outcomes are suggestive of effects secondary to paternal toxicity.

In summary, the assembled data support that the primary critical effects associated with the quats are point of contact effects, such as irritation and corrosion, which occur in a dose–response manner. Other effects, such as acute toxicity, systemic effects associated with repeated exposure, and carcinogenicity, were not supported. The data regarding quats and DART are inconsistent, but DART was identified for this case study as a potential effect of interest.

### Step 2b: Identification of alternative health-based exposure limits

Along with the identification of the critical effect(s), dose–response analysis is the identification and review of alternative health-based exposure limits (HBELs). These exposure recommendations and benchmarks have been developed using similar approaches applied to derive OELs but are not established as OELs. Like OELs, numerous governmental agencies and public health institutes develop HBELs. Table 4 provides a listing of the databases searched for HBELs.

**Table 4.** Sources of alternative health-based exposure limits.

Governmental agency or public health institute	HBEL	Reference
ATSDR	MRL	ATSDR (2018)
CA OEHHA	Reference exposure levels	CA OEHHA (2020b)
EPA IRIS	RfC	EPA (2020c)
ECHA	DNEL	ECHA (2020a)
NAC	AEGLs	EPA (2020d)
NIOSH	IDLH	NIOSH (2019b)
TCEQ	ESLs	TCEQ (2020)

TCEQ: Texas Commission on Environmental Quality; NIOSH: National Institute for Occupational Safety and Health; NAC: National Advisory Committee for the Development of Acute Exposure Guideline Levels for Hazardous Substances; ECHA: European Chemical Agency; EPA: Environmental Protection Agency; IRIS: Integrated Risk Information System; CA OEHHA: California Office of Environmental Health Hazard Assessment; ATSDR: Agency for Toxic Substance and Disease Registry; MRL: minimal risk level; RfC: reference concentration; DNEL: derived no effect level; AEGL: acute exposure guideline level; IDLH: immediately dangerous to life or health; ESL: effect-screening level.

For this case study, these databases were searched for HBELs for the disinfectant and the individual quats. Only ECHA (2020a) contained HBELs in the form of derived no effect levels (DNELs) for the individual quats except for ODDAC. These DNELs were intended for inhalation, long-term worker scenarios. Table 5 provides supplemental information on the DNELs in addition to a summary of the basis of the DNELs for the quats.

**Table 5.** Basis of the long-term worker derived no effect levels (DNELs) for the quats.

Reference	Ingredients	Most sensitive endpoint	Route (species)	Basis	PoD after route to route extrapolation	UF	Long-term DNEL (inhalation – worker)
ECHA (2020b)	ADBAC	Repeated dose toxicity	Oral (dog)	47.5 mg/kg bw/day (NOAEL)	23.75 mg/m^3^ (NOAEC)	6 (3× = intraspecies; 2× = interspecies toxicokinetics)	3.96 mg/m^3^
ECHA (2020c)	DDAC	Repeated dose toxicity	Oral (rat)	31 mg/kg bw/day (NOAEL)	54.6 mg/m^3^ (NOAEC)	3 (intraspecies)	18.2 mg/m^3^
ECHA (2020d)	Dio-DAC	Chronic/carcinogenicity	Oral (rat)	32 mg/kg bw/day (NOAEL)	56.39 mg/m^3^ (NOAEC)	3 (intraspecies)	18.79 mg/m^3^
ECHA (2020e)	ODDAC	N/A	N/A	N/A	N/A	N/A	N/A

ADBAC: alkyl (C_12_-C_16_) dimethyl benzyl ammonium chloride; DDAC: didecyl dimethyl ammonium chloride; Dio-DAC: dioctyl dimethyl ammonium chloride; DNEL: derived no effect level; NOAEC: no observed adverse effect concentration; NOAEL: no observed adverse effect level; PoD: point of departure; ODDAC: octyl decyl dimethyl ammonium chloride; UF: uncertainty factor.

**Figure 2.**
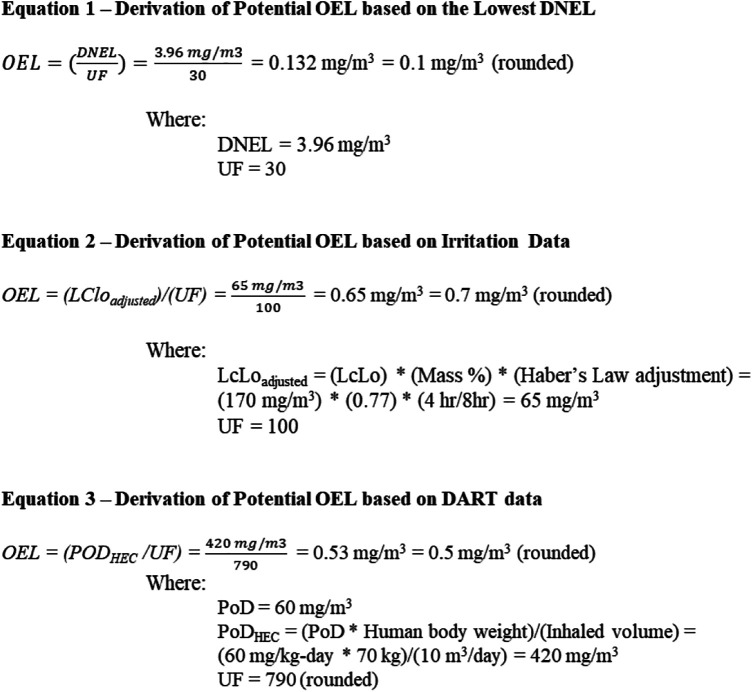
Derivation of potential OELs for quats based on identified lines of evidence. BW: human body weight; DNEL: derived no effect level; HEC: human equivalent concentration; LCLo: lowest concentration that caused lethality; PoD: point of departure; OEL: occupational exposure limit; UF: uncertainty factor.

In summary, this step is intended to facilitate the identification and review of alternative HBELs for the active ingredients that may serve as the basis of an OEL. In this case study, the long-term inhalation worker DNEL for ADBAC is the lowest among the identified DNELs. Modification of this DNEL is, therefore, described in the following section and in Figure 2 (equation (1)).

### Step 3: Development of potential OELs

Based on the information obtained during the toxicological screening assessment, derivation of OELs for the quats was conducted based on the three identified lines of evidence: (1) lowest worker DNEL, (2) irritation data, and (3) DART data. The following sections describe the rationale for the derivation of potential OELs for the quats via the different lines of evidence. Figure 2 provides the specific equations applied to derive the potential OELs for each line of evidence.

### Derivation of potential OEL based on lowest worker DNEL

This approach was based on modifying the lowest DNEL for the quats summarized in Table 5. The lowest DNEL was identified as 3.96 mg/m^3^ for ADBAC (ECHA, 2020b). This DNEL was based on a NOAEL selected from a repeat-dose oral dosing study in dogs. Following route-to-route extrapolation, an inhalation equivalent no-observed-adverse-effect concentration (NOAEC) was calculated at 23.75 mg/m^3^. The NOAEC was divided by a total UF of 6 to account for intraspecies and human variability resulting in the final worker DNEL for ADBAC (3.96 mg/m^3^).

To ensure that an OEL based on the lowest worker DNEL is health protective against the health effects of concern, including irritation, additional UFs were applied. As previously described, the DNEL was based on the oral data from a repeated dose toxicity study. This study did not report irritation, which is the primary adverse effect associated with inhalation exposures to quats. For this reason, the DNEL was divided by a total UF of 30, which reflects default values to account for database limitations (i.e. lack of inhalation data for irritation; 10×) and absence of a toxicodynamics adjustment in the original DNEL calculation (3×). This OEL based on the lowest worker DNEL has been calculated based on equation (1) and is 0.1 mg/m^3^.

### Derivation of potential OEL based on irritation data

This approach relies on deriving the OEL for the quat using risk assessment strategies intended to be protective against acute toxicological effects (i.e. irritation) (NAS, 2001; NIOSH, 2013). A PoD was selected from an acute inhalation toxicology study in which rats were exposed to ADBAC for 4 h (ECHA, 2020b). The lowest concentration that caused lethality (LCLo; 1/10 animals died) was 170 mg/m^3^ for ADBAC. It was further reported that ADBAC represented 77% of the test substance resulting in a mass-based equivalent concentration of 131 mg/m^3^. This value was adjusted to an 8-h exposure duration using Haber’s law equations, resulting in a concentration of approximately 65 mg/m^3^ (NAS, 2001; NIOSH, 2013). A total UF of 100 was applied to account for animal to human variability (10×), individual susceptibility (3×), and database limitations (3×). These UFs were selected based on the guidance outlined by NAS (2001) and NIOSH (2013). Equation (2) demonstrates the derivation of the potential OEL based on irritation data, which is 0.7 mg/m^3^.

### Derivation of potential OEL based on DART

This approach relies on deriving an OEL for quats intended to be protective specifically against DART in the event that such an effect could occur with exposure to these compounds. A repeated dose oral study in mice was identified that investigated DART effects associated with exposure to a disinfectant containing a mixture of two quats (ADBAC at 6.76% and DDAC at 10.1%) (Melin et al. 2014). The PoD was a minimal LOAEL of 60 mg/kg/day. When converted to a human equivalent concentration (HEC), as demonstrated in equation (3) in Figure 3, the PoD_HEC_ is 420 mg/m^3^. Based on ECHA (2008) and ECETOC (2014), a total UF of 790 (rounded) was applied to account for bodyweight adjustment from mice to humans (7×), toxicodynamics (2.5×), worker population (5×), database limitations (3×), and adjustment from a minimal severity LOAEL to NOAEL (3×). The OEL derived by this approach is 0.5 mg/m^3^. Equation (3) illustrates the calculation of the potential OEL via this approach. Note that since the standard guideline studies did not identify a DART hazard, this line of evidence is developed only to verify that DART would not be the critical effect for an OEL.

### Step 4: Selection of final OEL

The reported health hazards for the quat-based disinfectant identified in the product information and SDS were skin corrosion/irritation and serious eye damage/irritation. With the exception of ethanol, OELs were not available for the other active ingredients. There are also reports of DART in animal studies at doses that also cause systemic effects; however, the available animal data neither provide conclusive evidence as to whether quats can induce DART nor do they provide evidence of the potential toxicological mechanism for these effects. A review of alternative HBELs resulted in the identification of long-term inhalation worker DNELs for three of the quats (ADBAC, Dio-DAC, and DDAC); however, no DNELs or HBELs were available for ODDAC.

Three potential OELs were developed based on different lines of evidence, including toxicity data for irritation and DART, in addition to an alternative HBEL in the form of the lowest identified DNEL. The range of OELs calculated based on the previously described rationale and the equations presented in Figure 2 ranged from 0.1 mg/m^3^ to 0.7 mg/m^3^. The lowest OEL of 0.1 mg/m^3^ was derived by modifying the lowest long-term worker DNEL. It is anticipated that this OEL will be protective against the acute (i.e. irritation/corrosive) and DART effects that served as the basis of the other potential OELs for the quats (Figure 2, equations (2) and (3)) since the potential OELs derived based on these PoDs were above 0.1 mg/m^3^. Therefore, based on the available data, an OEL for this quat-based product of 0.1 mg/m^3^ is adequately protective and is intended to be applied as a time-weighted average (TWA) concentration for an 8-h workday.

### Summary

This framework is intended to guide users through the key considerations and overall process for the derivation of OELs for antimicrobial agents. The process relies on a weight-of-evidence approach that integrates data from multiple lines of evidence to identify critical effects, PoDs, and guides the selection of UFs to facilitate the derivation of OELs for an antimicrobial product or its active ingredients. The case study was conducted to demonstrate the application of the framework. The toxicity of the overall disinfectant was attributed to the quats. Although quats are well recognized for their ability to cause skin and eye irritation and damage under certain conditions, their ability to cause DART is not as well characterized. Overall, the toxicity data and supplemental guidance (i.e. DNELs) were sufficient to derive three potential OELs based on multiple different lines of evidence: (1) the lowest DNEL, (2) irritation data, and (3) DART data. The lowest OEL based on a DNEL derived in the case study was selected as the OEL for this quat-based antimicrobial product.

## Discussion

In response to the ongoing COVID-19 pandemic, guidance for cleaning and disinfecting of environmental surfaces has been issued that emphasizes the use of EPA-approved disinfectants (AIHA, 2020; CDC, 2020). Many of the active ingredients contained in these antimicrobial agents are recognized occupational health hazards that may cause a wide range of adverse effects (NTP, 2019a). Furthermore, patterns of product use and misuse may represent an important variable when considering OELs and relative human health risk. In the United States, poison control centers (PCCs) frequently respond to human exposures involving surface cleaners and antimicrobials and represent an important source of human exposure data. The American Association of Poison Control Centers (AAPCC, 2020) identified that 20,958 disinfectant exposure cases were reported to the 55 regional PCCs from January 1, 2020, through August 30, 2020, representing an increase of 55% compared to the same time period during the previous year. The majority of these exposure cases (approximately 90%) were unintentional in nature and help to further illuminate the broad spectrum of product use scenarios that may influence health risks in both occupational and nonoccupational settings. PCC data may help to further identify and prioritize active ingredients that do not have OELs and may benefit from our proposed framework. Additionally, these data may illuminate the conservativeness of OELs that are established in relation to exposure patterns as well as preventative steps that can be taken to reduce total exposure burden within various settings.

The case study described herein for a quat-based disinfectant illustrates the application of a framework to derive OELs for antimicrobial products. Based on the screening level assessment, the focus was placed on deriving an OEL for the quats, which are a family of chemicals recognized for their antimicrobial properties and are commonly used as active ingredients in disinfectants, sanitizers, and sterilants used for floors, countertops, or medical devices through various application process, such as spraying, wiping, and washing (LeBouf et al., 2017). In addition, quats-based disinfectants represent the overwhelming majority of disinfectants found in *List N*, and no existing OELs for the quats reviewed in the case study were identified.

The OEL of 0.1 mg/m^3^ derived through the case study is intended to be applied as a TWA concentration for an 8-h workday. For short-term or acute exposure scenarios, it is recommended to follow ACGIH TLV guidance for TLV-TWAs that do not have a TLV-STEL: “*Transient increases in workers’ exposure levels may exceed three times the value of the TLV-TWA level for no more than 15 min at a time, on no more than four occasions spaced 1 h apart during a workday, and under no circumstances should they exceed five times the value of the TLV-TWA level when measured as a 15-min TWA*” (ACGIH, 2019). In other words, exposure levels may exceed a level of 0.3 mg/m^3^ for 15 min or less, spaced at least 1 hour apart, on no more than four occasions during a workday; additionally, exposure levels should not exceed 0.5 mg/m^3^ on any occasion when measured as a 15-min TWA.

It is important to note that the OEL of 0.1 mg/m^3^ is intended to be applied for total quats in this mixture because it has been assumed that each quat is equally potent in toxicity based on the limited data. In the event that there are sufficient data for each quat to determine potency differences, the OEL could be modified to better reflect the relative toxicity of the individual quats. The modified OELs could then subsequently be applied using the mixture formula to calculate the hazard index, or the ratio of the exposures divided by the OELs, to assess exposures of the total mixture (ACGIH, 2019).

Another consideration that may need to be addressed when developing OELs for antimicrobial agents is the use scenario. These products, such as the quat-based disinfectant that was the subject of the case study, may be found in concentrated or diluted forms. The OELs developed in the case study were established based on data for exposure to concentrated forms of the quats. Since the critical effect is irritation, protection from adverse effects in concentrated form is equal to or more protective than exposures to dilute forms. Development of OELs for different dilutions is not practical since the dilution can change drastically for each use. Hence, OELs derived based on the concentrated form are considered protective.

For the derivation of potential OELs using the three lines of evidence outlined in Figure 2, the numeric value of the factor was applied using default values of one order of magnitude (i.e. 10×) or the square root of this value (i.e. 3.2× presented as 3×). Use of such defaults has the advantage of simplicity for a screening limit and is consistent with typical UF approaches in the regulatory context. Additional research and analysis of the mode of action considerations might inform refined factors. One methodology for refinement is the Chemical Specific Adjustment Factor (IPCS, 2005). However, data are not adequate for the quats to apply this methodology in the case study. For example, specific data of variability of human response to inhalation exposures to quats were not identified. Several considerations suggest that the default UF values applied might be more than adequately protective. For example, a UF value of 10× was used for database insufficiency due to the lack of an inhalation study and repeated exposure PoD for direct respiratory effects for modification of the DNEL. Since quats are direct-acting irritants, the effects in the oral dosing study that was used as the basis of the DNEL likely already reflected some degree of direct tissue reactivity. However, the use of a full factor of 10 is supported by the resulting OEL that is similar in value to the OEL derived directly from the acute inhalation study (see equation (1) in Figure 2). Additional quantitative data on respiratory tract irritant potency would resolve this data gap. The worker DNEL also incorporated a factor of 3× for human variability in sensitivity. Although this factor is lower than the full 10× value used for the general population, and even lower than the typical 5× employed for worker risk assessment in the DNEL guidelines, further reduction might be warranted based on mode of action considerations. For direct reacting chemicals, some assessments have incorporated a reduced factor (NIOSH, 2013). The use of a 3× factor was employed since no factor for toxicodynamics was incorporated into the DNEL (since it was derived based on an oral study). Based on the mode of action of direct tissue reactivity, a reduced factor could also be considered. Together, the OEL of 0.1 mg/m^3^ derived from the oral toxicity study and represented in the inhalation DNEL with an additional 30× composite factor likely is more than adequately protective. However, this is an argument made based on general principles for direct-reacting irritants, and chemical-specific data are not available.

Although not directly highlighted in this manuscript, read-across methods and tools could be integrated into the framework. These methods are intended to assist in characterizing the adverse effects associated with a chemical of interest through data for other chemicals that cause toxicity via the same mode of action because of similar structures and physiochemical properties (Escher et al., 2019). For example, the disinfectant highlighted in the case study was composed of four quats that share a functional group that is responsible for the toxicity of the chemical class. Despite that the majority of the identified data were for ADBAC and DDAC, the limited data for the other quats (i.e. Dio-DAC and ODDAC) were useful because of their structural and physiochemical similarities in characterizing the overall toxicity of the disinfectant. Formal application of read-across, especially those that include in vitro and computational considerations, could further enhance the usefulness of the framework for deriving OELs for antimicrobial agents with active ingredients, such as quats, alcohols, or aldehydes that have a common functional group that is responsible for the antimicrobial activity and subsequent toxicity.

As a point of comparison, it is interesting to note that NIOSH (2019a) has developed a process called occupational exposure banding (OEB) to assign chemicals without OELs into specific categories or bands that correspond to a range of exposure conditions intended to protect worker health. This process is divided into three distinct tiers that are intended to band a chemical based on the available technical information. The lowest tier relies on only the GHS hazard statements (H-codes) listed on a SDS for a chemical. In regard to the case study presented in this article, a tier 1 assessment was conducted using a screening-level assessment e-tool developed by NIOSH for the individual quats. This assessment was conducted to compare the potential OELs derived to the case study to alternate recommendations generated via other data sources. The collective results of the tier 1 assessment classified the overall disinfectant and individual quats in the most severe category (i.e. band E), which aligns with an airborne concentrations of <0.1 mg/m^3^ (NIOSH, 2019a, c) and the lowest potential OEL derived in the case study. The tier 1 assessment resulted in a similar but more health protective recommendation than the framework, which is anticipated for a screening-level assessment tool like the NIOSH OEB e-tool.

## Conclusion

Antimicrobial products are routinely relied upon to ensure food and drug safety, prevent hospital-acquired infections, and treat drinking and wastewater, in addition to various other industrial, commercial, and residential applications. However, while many of the active ingredients in such products also represent health hazards, existing OELs for these chemicals are limited. A framework was developed to be used by trained occupational health professionals to derive OELs for antimicrobial agents. As a demonstration of the application of this framework, a case study for a disinfectant product containing quats was presented. A weight-of-evidence approach was used to derive three potential OELs for quats based on irritant toxicity, DART, and modified existing HBEL. A final OEL of 0.1 mg/m^3^ was selected for quats and is considered to be protective of all potential identified adverse health outcomes.
